# Health Utilities of Type 2 Diabetes-Related Complications: A Cross-Sectional Study in Sweden

**DOI:** 10.3390/ijerph110504939

**Published:** 2014-05-07

**Authors:** Aliasghar A. Kiadaliri, Ulf-G Gerdtham, Björn Eliasson, Soffia Gudbjörnsdottir, Ann-Marie Svensson, Katarina Steen Carlsson

**Affiliations:** 1 Health Economics Unit, Department of Clinical Sciences, Lund University, Lund 22381, Sweden; E-Mails: ulf.gerdtham@med.lu.se (U.-G.G.); katarina.steen.carlsson@med.lu.se (K.S.C.); 2 School of Public Health, Department of Health Management and Economics, Tehran University of Medical Sciences, Tehran 141556447, Iran; 3 Institute of Economic Research, Health Economics & Management, Lund University, Lund 22007, Sweden; 4 Department of Economics, Lund University, Lund 22363, Sweden; 5 Sahlgrenska University Hospital, Department of Medicine, University of Gothenburg, Gothenburg 41345, Sweden; E-Mails: bjorn.eliasson@gu.se (B.E.); soffia.gudbjornsdottir@medic.gu.se (S.G.); Ann-Marie.Svensson@registercentrum.se (A.-M.S.)

**Keywords:** EQ-5D, health utility, Sweden, type 2 diabetes

## Abstract

This study estimates health utilities (HU) in Sweden for a range of type 2 diabetes-related complications using EQ-5D and two alternative tariffs (UK and Swedish) from 1757 patients with type 2 diabetes from the Swedish National Diabetes Register (NDR). Ordinary least squares were used for statistical analysis. Lower HU was found for female gender, younger age at diagnosis, higher BMI, and history of complications. Microvascular and macrovascular complications had the most negative effect on HU among women and men, respectively. The greatest decline in HU was associated with kidney disorders (−0.114) using the UK tariff and stroke (−0.059) using the Swedish tariff. Multiple stroke and non-acute ischaemic heart disease had higher negative effect than a single event. With the UK tariff, each year elapsed since the last microvascular/macrovascular complication was associated with 0.013 and 0.007 units higher HU, respectively. We found important heterogeneities in effects of complications on HU in terms of gender, multiple event, and time. The Swedish tariff gave smaller estimates and so may result in less cost-effective interventions than the UK tariff. These results suggest that incorporating subgroup-specific HU in cost-utility analyses might provide more insight for informed decision-making.

## 1. Introduction

Diabetes mellitus is a chronic disease with a growing worldwide prevalence. The International Diabetes Federation estimated that 366 million people had diabetes in 2011, and it is predicted that this figure will undergo a 51% increase by 2030 [1]. Type 2 diabetes accounts for 90–95% of the incidence of diabetes [2], and is associated with a higher risk of macro- and microvascular complications [3,4]. A pooled analysis [5] based on 8.49 million person-years at risk showed that hazard ratios of coronary heart disease, ischaemic stroke, and haemorrhagic stroke were 2, 2.27, and 1.56 for diabetics *versus* non-diabetics, respectively. The Framingham Heart Study revealed that the 30-year risk of developing cardiovascular disease was significantly higher among men and women with diabetes compared to those without diabetes [6]. Moreover, among patients with diabetes, experiencing a first event substantially elevates the risk of subsequent events [7].

This association between diabetes and complications means that people with diabetes not only have shorter life expectancy than people without diabetes [8,9], but also experience poorer health-related quality of life (HRQoL) than the general population [10,11,12]. Moreover, patients with diabetes-related complications have lower HRQoL than diabetes patients without complications [13,14,15]. It is essential to consider this impact on HRQoL in health economic models, since subjective health status is an important aspect of cost-effectiveness of treatments. From a health economics perspective, a preference-based measure of HRQoL is required to estimate health-state utility values and calculate quality-adjusted life years (QALYs). 

Clinical, environmental and organizational disparities across countries might limit transferability of results of studies on health utility (HU) [14]. In other words, the same clinical or sociodemographic variable might have a different impact on HU in different settings. For example, in one geographical area, patients might receive sufficient care to manage their diabetes, resulting in a higher HU compared with an area where this is not the case. Therefore, eliciting and incorporating country-specific health utilities for different health states is necessary to assess the effectiveness and cost-effectiveness of interventions and improve the relevance of analyses supporting informed decision-making in health care. 

While HU scores for a range of common diabetes-related complications among patients with type 2 diabetes are available for other countries [14,15,16,17], there is limited information on these scores in Sweden. Previous Swedish studies have reported results on the impact of a specific complication on HU [18,19], or included only type 1 diabetes patients [20]; others have evaluated the association between complications and HRQoL rather than HU [21,22], which limits their application in economic analyses. 

The EQ-5D instrument is based on five attributes, and the responses to these attributes are weighted based on the preferences elicited from a sample of general population/patients to calculate an index score. Until recently, Swedish studies generally applied the UK tariff [23] because no Swedish tariff existed. However, a Swedish experience-based tariff for the EQ-5D was recently published [24]. 

The primary aim of this study was to estimate health utilities associated with a range of type 2 diabetes-related complications using survey data on EQ-5D responses and registry data on complications among a large sample of people with type 2 diabetes from the Swedish National Diabetes Register (NDR). We also aimed to investigate heterogeneities in the effect of complications on HU in terms of gender, multiple event, and time since event. Both the UK and Swedish tariffs were used, and the results were compared. In the current study, we applied the UK tariff in the main analysis and the Swedish tariff as a sensitivity analysis. One of the main reasons for this was that the UK tariff is the most common tariff used in the literature, which increases comparability of our study with previous studies. Another major reason was that there are other diabetes-related complications (e.g., neuropathy) which we did not include in the current study due to lack of data; this implies that the HU of these missing complications should be extracted from previous studies, which mainly applied the UK tariff. 

## 2. Method and Materials

### 2.1. Setting and Participants

The NDR was established to follow up and improve the quality of diabetes care in Sweden [25]. It includes individual-level demographic and clinical data (e.g., risk factors, treatment, diabetes-related complications) on adult individuals aged ≥18 years that have agreed to be registered before inclusion. Participation in the NDR is not compulsory, and patients are offered the option of being excluded if they wish. Data are reported to the NDR from all hospital diabetes outpatient clinics and primary health care centres at least once a year. 

In order to improve knowledge about the quality of diabetes care in Sweden, the NDR conducted a survey in 2008 to collect data on patients' HRQoL using the Swedish version of EQ-5D (IQ3 project). Twenty-six primary health care centres participated in the IQ3 project. All patients who visited one of these centres during the recruitment period (1 February to 30 May 2008) were selected to participate, as long as they met the inclusion criteria: (1) aged 18–80 years; (2) time since diagnosis longer than 6 months; and (3) not living under a protected identity. A total of 4,760 patients with type 1 or type 2 diabetes met these criteria, and thus were mailed the Swedish version of the EQ-5D questionnaire between June and August 2008. Of these, 3,089 questionnaires were returned by 2947 patients (there were 115 patients with duplicates responses), giving a response rate of 63%. The current study included 1757 patients who met the additional inclusion criteria: (1) type 2 diabetes (treatment with diet or oral hypoglycaemic agent (OHA) only regardless the age at onset of diabetes, or treatment with insulin alone or in combination with OHA and age ≥40 years at onset of diabetes); (2) data available on history of diabetes-related complications; and (3) age at diagnosis older than 25 (Figure S1 in the Supplementary Material). There were significant differences by age, age at diagnosis and duration of diabetes between included (n = 1757) and excluded (n = 170) patients, but no significant difference in term of BMI, gender and EQ-5D scores (Table S1 in the Supplementary Material). 

### 2.2. Utility Measurement

EQ-5D is most used multi-attribute instrument worldwide for eliciting health-related preferences. It is based on five attributes: mobility, self-care, usual activities, pain/discomfort, and anxiety/depression. Each attribute has three levels: no problems, some problems, and severe problems [26], resulting in 243 (3^5^) possible health states. In the current study, both UK [23] and Swedish [24] tariffs were applied to calculate EQ-5D index scores, and the results of applying these tariffs were compared. 

### 2.3. Diabetes-Related Complications

Diabetes-related complications were defined in terms of International Classification of Disease, 10th revision (ICD-10) codes:

(1) Acute myocardial infarction (AMI): I21;
(2) Heart failure (HF): I50;
(3) Non-acute ischaemic heart disease (NAIHD): I22, I24.8, and I24.9 including stable and unstable angina (I20.0, I20.1, I20.8, and I20.9);
(4) Stroke: I61, I63, I64, and I67.9,
(5) Kidney disorders: N00-N08, N10-N16, N28.9, E11.2, E14.2, Z49.1, Z49.2, Z99.2, Z94.0, N17, N18, N19;
(6) Retinopathy: H35.0, H35.2, H35.6, H35.9, H36.0, E11.3;
(7) Amputation: ankle (S98.0), lower leg (S88), hip (S78.0), and pelvis (S38.3).

These events were retrieved by data linkage with the Swedish Cause of Death and Hospital Discharge Registers. In this study, we considered any episode of hospitalization as an event (e.g., two hospitalization with diagnostic code of I50 were considered as two heart failures). 

In all analyses, these complications were included in two forms. Model 1 included pooled events with AMI, HF, NAIHD, and stroke classed as macrovascular complications; and kidney disorders, retinopathy, and amputation classed as microvascular complications. Model 2 focused on specific events, with each event included as a separate event. It should be noted that in the latter model, history of amputation was not included due to low number of events. Earlier studies have suggested that age, gender, duration of diabetes, body mass index (BMI), and treatment modality are additional predictors of HRQoL among patients with type 2 diabetes [14,16,17], and so these aspects were included in our analyses.

### 2.4. Statistical Analysis

The continuous variables are presented as mean and standard deviation. The categorical variables are expressed as percentages. Ordinary least squares (OLS) regression with robust standard errors was applied to model EQ-5D index score. Due to the skewed distribution of the EQ-5D data, several different methods have been applied to these data in the literature [27,28,29], but OLS regression is the most common. Moreover, it can be argued that from a health economic perspective where HU is the main interest of analysis, OLS with robust standard errors is a valid approach [29]. The linearity of the continuous variables was checked using design variables and residual plots. To check gender heterogeneities in effect of complications, we estimated gender-specific models and used the *suest* command in STATA 13 (StataCorp LP, College Station, TX, USA) to compare coefficients between men and women. This command simultaneously estimate different models at once and combines the estimation results into one parameter vector and a simultaneous (co-)variance matrix of the sandwich/robust type; its main application is cross-model hypothesis tests. After this, the *test* command is used to get Wald test for equality of coefficients between models. While, in our study the same analysis could have been done using interactions terms, but *suest* avoid to fit a model with twice the number of coefficients (an interaction term with gender for all covariates). However, to keep our estimates comparable with previous studies and to avoid instability of regression coefficients due to low numbers of some diabetes-related complications, we answered the remaining research questions using the total sample. Continuous variables were treated as mean-centred values. The Wilcoxon matched-pairs signed-rank test was used to compare the median and distribution of the UK and Swedish tariffs, and Spearman’s rank correlation was used to examine the consistency between these tariffs in their ranking of observed health states.

## 3. Results

### 3.1. Descriptive Statistics

The mean age at diagnosis was 56.5 (±9.4) and the mean duration of diabetes was 9.5 (±7.1) years. Table 1 presents sample characteristics divided by gender. 

**Table 1:** Patients’ demographic and clinical characteristics divided by sex.

Variable	Men	Women
N	997	760
Age at diagnosis, years	55.65 ± 9.04	57.66 ± 9.75
Diabetes duration, years	9.94 ± 7.30	9.02 ± 6.92
BMI	29.46 ± 4.71	30.16 ± 5.95
Treatment		
Diet	12.04	17.76
OHA	36.31	37.37
Insulin ± OHA	51.65	44.87
History of acute myocardial infarction (%)	13.14	5.92
History of stroke (%)	7.72	4.87
History of heart failure (%)	5.42	3.95
History of non-acute ischaemic heart disease (%)	16.35	8.03
History of retinopathy (%)	2.31	1.97
History of kidney problems (%)	2.71	2.63
History of amputation (%)	0.70	0.53

Insulin with or without oral hypoglycaemic agents was the most frequent treatment among both women and men. The prevalence of diabetes-related complications was higher among men than among women. NAIHD was the most common diabetes-related complication, and amputation was the least common.

The distribution of “some or severe problems” in the EQ-5D attributes is given in Table 2. Among the EQ-5D attributes, the highest prevalence of “some or severe problems” was reported in pain/discomfort (55.5%) and the lowest in self-care (5.5%). Women reported a higher proportion of “some or severe problems” in four out of five attributes of the EQ-5D (*p* < 0.001). There was no gender difference in the proportion rating a negative UK EQ-5D score, signalling a health state worse than death (2.0% of men and 1.8% of women; *p* = 0.81). On the other hand, 41% of men and 25% of women reported no problems in any attribute of EQ-5D; that is, UK EQ-5D score = 1 (*p* < 0.001).

**Table 2:** Percentage of respondents with moderate/severe problems in EQ-5D dimensions and EQ-5D score.

EQ-5D Dimension	Women	Men	Total
Mobility			
Moderate	31.84	26.48	28.80
Severe	0.26	0.40	0.34
Self-care			
Moderate	4.21	4.21	4.21
Severe	0.66	1.81	1.31
Usual activities			
Moderate	19.61	15.05	17.02
Severe	1.84	2.41	2.16
Pain/discomfort			
Moderate	55.79	44.03	49.12
Severe	7.11	5.82	6.37
Anxiety/depression			
Moderate	41.97	29.19	34.72
Severe	3.55	2.71	3.07
EQ-5D index score (UK tariff) (95%CI)	0.74 (0.72–0.76)	0.79 (0.77–0.80)	0.77 (0.75–0.78)
EQ-5D index score (Swedish tariff) (95%CI)	0.86 (0.86–0.87)	0.88 (0.88–0.89)	0.88 (0.87–0.88)

### 3.2. Regression Analyses

#### 3.2.1. Pooled Sample Analysis

When the UK tariff was used in the total sample (Table 3, columns 7 and 8), younger age at diagnosis was associated with lower HU scores (marginally significant). BMI had a negative effect on HU scores; a BMI increase of 5 units was associated with a decrease of 0.03 in HU. Women had lower HU scores than men. Interestingly, Model 1 (pooled events) showed that micro- and macrovascular complications had the same negative impact on HU scores. Specific event analysis revealed that kidney disorders had the most negative impact on EQ-5D scores in the total sample.

**Table 3:** Factors associated with variation in EQ-5D score: results from the UK tariff.

Variable	Women		Men		Total	
	Model 1	Model 2	Model 1	Model 2	Model 1	Model 2
Constant	0.7527 ***	0.7478 ***	0.8357 ***	0.8347 ***	0.8205 ***	0.8167 ***
Female gender	NA	NA	NA	NA	−0.0570 ***	−0.0561 ***
Age at diagnosis	0.0012	0.0012	0.0017	0.0019 *	0.0015 *	0.0015 *
Diabetes duration	−0.0010	−0.0015	−0.0010	−0.0007	−0.0010	−0.0010
BMI	−0.0066 ***	−0.0065 ***	−0.0054 ***	−0.0053 ***	−0.0060 ***	−0.0060 ***
History of macrovascular events	−0.0488 **		−0.1008 ***		−0.0831 ***	
History of microvascular events	−0.1603 ***		−0.0344		−0.0830 **	
AMI history		−0.0032		−0.0345		−0.0220
Stroke history		−0.0610		−0.1395 ***		−0.1111 ***
HF history		−0.0506		−0.1049 **		−0.0821 **
NAIHD history		−0.0463		−0.0547 **		−0.0516 **
Kidney disorders history		−0.2482 ***		−0.0110		−0.1144 **
Retinopathy history		0.0026		−0.0304		−0.0119
Treatment						
Diet (ref)						
OHA	0.0158	0.0210	−0.0210	−0.0209	−0.0014	0.0010
Insulin ± OHA	−0.0046	0.0016	−0.0189	−0.0188	−0.0086	−0.0055
R-squared	0.06	0.07	0.05	0.06	0.06	0.07
N	760	760	997	997	1757	1757

*,**,***: significant at 10%, 5%, and 1%, respectively.

#### 3.2.2. Multiple Events and Time Since Event

Table 4 presents the effect of single and multiple events on the UK HU scores in the total sample. Model 1 (pooled events) showed no significant differences in HU scores between patients with single and multiple events, after controlling for other covariates. However, the event-specific model (Model 2) showed that patients with multiple stroke and NAIHD had significantly lower HU than patients with a single event.

#### 3.2.3. Gender-Specific Analysis

The results of the OLS analysis of UK EQ-5D index scores among women and men are presented in Table 3. Model 1 showed that the highest negative effect on HU scores came from history of microvascular complications among women and history of macrovascular complications among men. Cross-model comparisons of Model 1 showed that the coefficients for micro- and macrovascular complications were different among men and women (marginally significant; *p* = 0.05 for microvascular and *p* = 0.09 for macrovascular complications). Model 2 showed that among women, kidney disorders had the highest negative effect on HU and none of the macrovascular complications were significantly associated with HU. On the other hand, among men, stroke had the highest negative effect on HU and there were no significant associations between microvascular complications and HU scores. Across Model 2, the estimates on kidney disorders were significantly different among men and women (*p* = 0.01). Neither model showed any significant differences in HU scores among patients in different treatment groups.

**Table 4:** Effect of single and multiple events on EQ-5D score: results from the UK tariff.

Variable	Model 1		Model 2	
	Coefficients ^a^	Equality of coefficients ^b^	Coefficients ^a^	Equality of coefficients ^b^
Macrovascular events				
No (ref)				
Single	−0.0555 **	0.47		
Multiple	−0.1008 ***			
Microvascular events				
No (ref)				
Single	−0.0608	0.11		
Multiple	−0.1055 **			
AMI				
No (ref)				
Single			−0.0205	0.96
Multiple			−0.0230	
Stroke				
No (ref)				
Single			−0.0797 ***	0.06
Multiple			−0.2617 ***	
NAIHD				
No (ref)				
Single			−0.0021	0.04
Multiple			−0.0756 ***	

**,***: significant at 5%, and 1%, respectively. ^a^ Additionally adjusted for gender, age at diagnosis, BMI, duration of diabetes, treatment, history of heart failure, retinopathy, and kidney disorders. ^b ^We used the *test* command in STATA to check if the estimated coefficients were significantly different.

We also examined whether the effect of complications on HU varied with time since event (Table S2 in Supplementary Material). In Models 1 and 2, where we only included patients with a history of complications, we found that each year elapsed since micro- and macrovascular complications was associated with an increase in HU scores of 0.013 and 0.007 units, respectively. On the other hand, in the total sample (Models 3 and 4), we found that except for HF, HU scores did not change with time since event. 

### 3.3. Application of the Swedish Tariff

The mean (median) UK and Swedish EQ-5D scores were 0.77 (0.80) and 0.88 (0.91), respectively. We observed 75 health states out of 243 possible health states among our study sample. There were significant differences between tariffs regarding mean, median, and distribution (*p* < 0.001). However, Spearman’s rank correlation between the UK and Swedish tariffs in ranking observed health states was high (*p* = 0.87; *p* < 0.001). 

Tables S3 to S5 in the Supplementary Material show the results of regression analysis on the Swedish EQ-5D score. While the magnitudes of the estimated coefficients were smaller than the UK tariff estimates, the directions of associations were similar. The main differences when applying the Swedish tariff were that stroke was a significant predictor among women (Table S1, column 4); there was no gender-heterogeneity in the effect of macrovascular complications on HU scores across Model 1; the rank of complications in term of utility decrement was different (e.g., the greatest decline in HU score was associated with stroke using the Swedish tariff and kidney disorders using the UK tariff); and the estimate on microvascular complications in Model 2 of Table S3 was no longer statistically significant. 

## 4. Discussion

To assist cost-utility analyses for type 2 diabetes in Sweden, we estimated HU scores for a range of diabetes-related complications using data collected from a large cross-sectional sample in the Swedish National Diabetes Register (NDR). As expected, history of diabetes-related complications was associated with lower HU scores after controlling for other clinical and demographic covariates. In addition, we found heterogeneities in the effect of diabetes-related complications on HU scores in terms of gender, multiple events, and time since event. Applying the UK and Swedish tariffs resulted in discrepant estimates, meaning that different HU decrements would be used in cost-utility analyses which potentially might lead to divergent funding decisions.

The effects of diabetes-related complications on HU scores differed between men and women. Generally, there was more negative effect of microvascular complications among women and macrovascular complications among men. Previous studies have also reported heterogeneity in response to health measures depending on the respondents’ personal characteristics including gender [30,31,32]. This indicates that men and women respond differently to factors related to their health; this should be noted by clinicians, policy-makers, and researchers, and should be taken into account in economic evaluations of interventions. 

The more profound effect of multiple events compared with a single event for stroke and NAIHD implies that separating the first and subsequent events instead of pooling them as history of events would improve the accuracy of health economic simulation models. For example, if intervention A is more effective than intervention B for preventing the occurrence of multiple stroke events, then if an assessment fails to distinguish between number of events as predictors of HU, it will underestimate the value in QALY gains of intervention A and the results will be biased in favour of intervention B, all else being equal. 

We found that the HU decrement associated with micro- and macrovascular complications diminished over time among patients with a history of complications. This finding was expected, as it is believed that these events have a large detrimental effect on HRQoL during the time immediately following the event [33]. These results should be interpreted with caution, as the sample sizes for these analyses were low, especially for microvascular events. However, in the total sample, in line with results from the UKPDS 62 study [17], we found that the effect of complications on HU generally did not change with time since event. Further analyses in large samples of type 2 diabetes patients with a history of complications are required to better explain the effects of these complications on HU over time. 

Our results and knowledge from previous studies suggest that BMI has both a direct effect on HU in patients with type 2 diabetes and an indirect effect through its effect on developing diabetes-related complications. The HU decrement associated with a 5-unit difference in BMI was 60% (UK tariff) and 54% (Swedish tariff) of the effect of a NAIHD event. The estimated HU decrement per unit of BMI in our study (0.0060) was similar to estimates from previous studies (0.0061 and 0.0065) [16,34]. 

The mean UK EQ-5D index for patients with no complications (0.79) was similar to the value reported in the UKPDS 62 study (0.79) [17], but lower than the value reported for people with type 2 diabetes in Norway (0.85) [35]. On the other hand, the mean UK EQ-5D index for patients with complications in our study (0.70) was comparable to the value reported in both UKPDS 62 [17] and Norwegian studies [35] (0.73). In comparison with the Swedish general population [36], while the mean UK EQ-5D index was numerically lower in our study (0.77 *vs.* 0.84), these values approached each other with increasing age. When we used the Swedish tariff, these figures were 0.89 for patients with no complications and 0.84 for patients with complications, implying that the Swedish tariff resulted in significantly higher HU scores than the UK tariff.

The range of HU decrement for complications in the total sample varied from 0.012 to 0.114 using the UK tariff and from 0.010 to 0.059 using the Swedish tariff. Our estimates using the UK tariff were similar to previous studies in Canada (range: 0.046–0.102) [15] and the USA (range: 0.012–0.108) [14]. Despite this similarity in range of HU decrement, the estimated HU scores for specific complications in our study differed from previous studies. Differences in sample characteristics, clinical setting, range of complications included in the study, and methods for statistical analysis might have contributed to disparities in HU scores. 

Differences between the estimates from the Swedish and UK tariffs were likely due to the lower spread of the Swedish tariff. Discrepancy in rating approach (experienced health in the Swedish tariff *vs.* hypothetical health in the UK tariff) might be one reason for the difference in the observed range of HU scores. Previous studies have generally reported that the experienced health approach resulted in higher values than the hypothetical health approach [37,38]. Failure to rate the same health state, to have different measuring rods, and patient adaptation to a health state are among the main factors contributing to these discrepancies [39]. In line with a previous study [40], we found that with the hypothetical health approach, self-care and pain/discomfort were the most important attributes and usual activities was the least important. Conversely, with the experienced health approach, usual activity was the most important attribute and self-care was the least important. While these differences did not have a significant effect on the direction of the association between HU scores and complications, it is expected that the lower spread of the Swedish tariff will lead to smaller effect differences and in turn larger incremental cost-utility ratios in cost-utility analyses, implying less cost-effective interventions [41]. 

The availability of longitudinal data on diabetes-related complications from register data for a large sample of people with type 2 diabetes is a major strength of the current study, compared with previous studies which used self-reported data prone to recall bias. In addition, this data gave us the opportunity to investigate the effects of multiple events and time since event on patients’ HU, which many previous studies did not examine due to lack of relevant data. Furthermore, to our knowledge, this is one of the earliest applications of the Swedish tariff to estimate HU decrement in a Swedish sample. Providing estimates based on both the UK and the Swedish tariff makes it easier to conduct sensitivity analysis in future cost-utility analyses, as recommended by the Dental and Pharmaceutical Benefits Agency (TLV) in Sweden. 

The results of this study should be interpreted in light of some limitations. Firstly, we measured HU scores with the EQ-5D, which according to previous studies might not be able to discriminate between complications or treatment modality in a diabetes context [42,43]. For example, Kontodimopoulos *et al.* [43] found that EQ-5D could not discriminate between patients with and without diabetic retinopathy. This might partly explain the non-significant impact of some complications in our study. Secondly, it is believed that severity of diabetes-related complications has an important effect on patients’ HU [44]. We were not able to control for this, due to lack of data; this might be another reason for the lack of significant effect of certain complications (e.g., MI) on HU in our study. Thirdly, we did not control for some other comorbidities, again due to lack of data. Fourthly, the EQ-5D data were collected using a mail survey that is prone to selection bias when the response is not complete (e.g., healthier patients responded to the questionnaire). However, the 65% response rate was a reasonable one to investigate our research questions. Finally, this was a cross-sectional study, and so no causal inference should be drawn from the results. 

## 5. Conclusions

We have estimated HU scores for a range of demographic and clinical features of patients with type 2 diabetes. As expected, diabetes-related complications were associated with lower HU scores. We found evidence suggesting presence of heterogeneities in association between a number of diabetes-related complications and HU scores in terms of gender, multiple event and time since event. We suggest that these heterogeneities should be further investigated in studies with larger sample sizes as capturing these heterogeneities might improve the precision of cost-utility analyses in health care. In addition, using these HU weights may assist informed decisions by policy-makers in Sweden. Applying estimates from the Swedish tariff could result in less cost-effective interventions compared with the UK tariff, due to its lower spread. Using diabetes-specific measures alongside EQ-5D and evaluating HU at multiple points in time are topics for future studies. 

## Supplementary Files

Supplementary File 1 Supplementary Information (PDF, 141 KB)
