# Supplementary material for: Health Utilities of Type 2 Diabetes-Related Complications: A Cross-Sectional Study in Sweden
Source: Int J Environ Res Public Health. 2014 May 7;11(5):4939–52. doi: 10.3390/ijerph110504939 (PMC4053909; doi:10.3390/ijerph110504939)
Supplement: Supplementary File 1 — Supplementary Information (PDF, 141 KB) [file ijerph-11-04939-s001.pdf]

Supplementary Information

Health Utilities of Type 2 Diabetes-Related Complications: A Cross-Sectional Study in Sweden

Figure S1. Flow diagram of sample selection in the study.

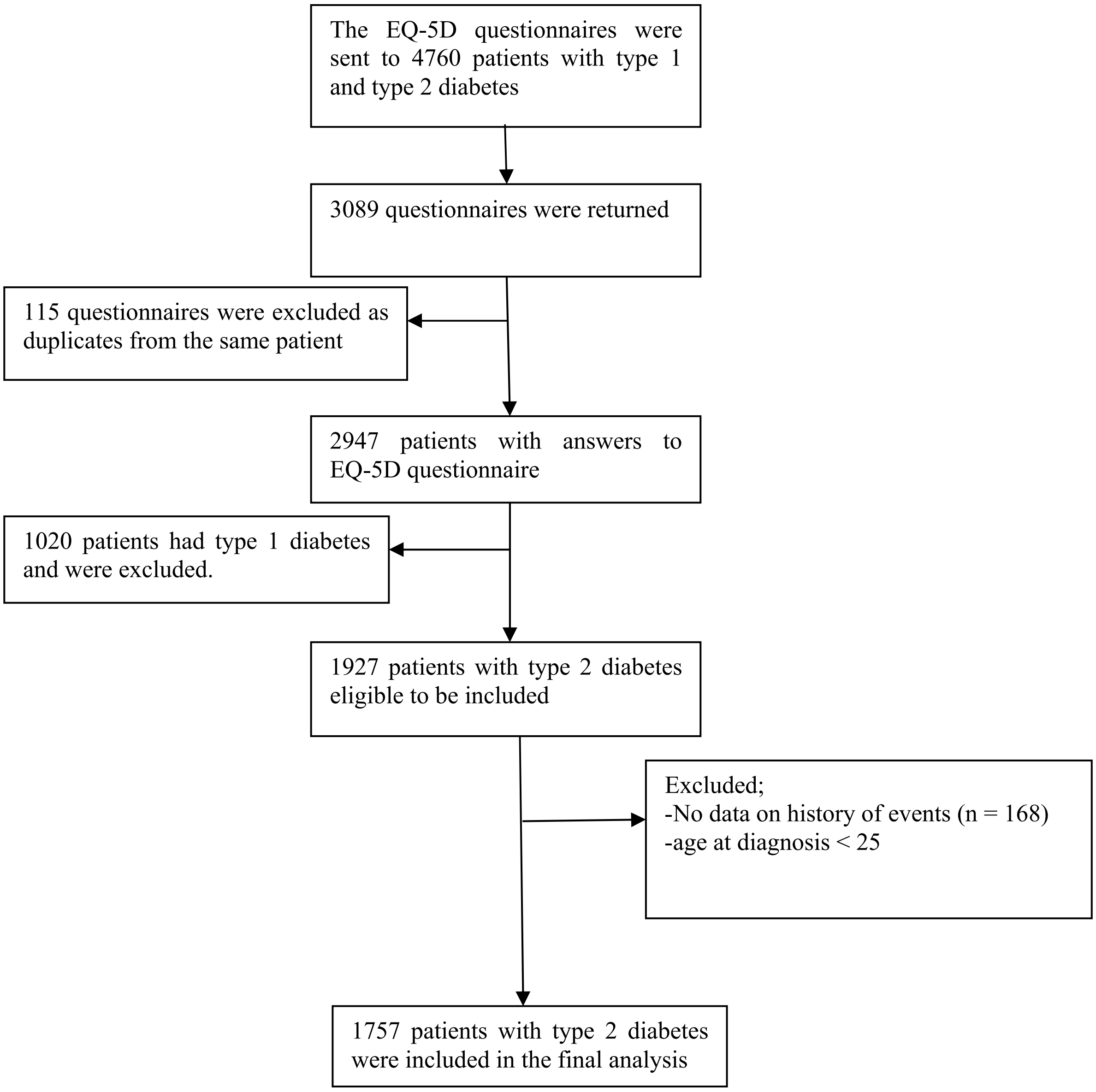

Table S1. Comparison of included and excluded subjects in terms of baseline characteristics.

Variable	Included (n = 1757)	Excluded (n = 170)	*p* Value of Difference
Age	66.1 (8.8)	64.0 (11.0)	0.004
Age at diagnosis	56.5 (9.4)	58.4 (11.8)	0.02
Diabetes duration	9.5 (7.1)	5.2 (7.9)	<0.001
Women (%)	43.3	48.2	0.21
BMI	30.0	30.0	0.71
EQ-5D index score	0.77 (0.3)	0.78 (0.3)	0.58

Table S2. Effect of diabetes-related complications on EQ-5D scores over time: results from the UK tariff.

Variable	Model 1	Model 2	Model 3		Model 4	
	Coefficients [a]	Coefficients [b]	Coefficients [c]	Equality of Coefficients [d]	Coefficients [c]	Equality of Coefficients [d]
Time since last macrovascular event						
Continuous (year)	0.0066 ***					
No event (ref)						
≤1 year			−0.0792 ***	0.18		
2−5 years			−0.1203 ***			
>5 years			−0.0517 **			
Time since last microvascular event						
Continuous (year)		0.0134 *				
No event (ref)						
≤1 year			−0.0978 *	0.91		
2−5 years			−0.0884			
>5 years			−0.0679			
Time since last AMI						
No event (ref)						
≤1 year					0.0674	0.13
2−5 years					0.0025	
>5 years					−0.0314	
Time since last stroke						
No event (ref)						
≤1 year					−0.0962 **	0.18
2−5 years					−0.2212 ***	
>5 years					−0.0708	
Time since last HF						
No event (ref)						
≤1 year					−0.0128	0.03
2−5 years					−0.2193 ***	
>5 years					−0.0296	

Table S2. *Cont.*

Variable	Model 1	Model 2	Model 3		Model 4	
	**Coefficients [a]**	**Coefficients [b]**	**Coefficients [c]**	**Equality of Coefficients [d]**	**Coefficients [c]**	**Equality of Coefficients [d]**
Time since last NAIHD						
No event (ref)						
≤1 year					−0.0717 **	0.80
2−5 years					−0.0622 *	
>5 years					−0.0451	
Time since last kidney disorders						
No event (ref)						
≤1 year					−0.1624 **	0.50
2−5 years					−0.1629 **	
>5 years					−0.0656	
Time since last retinopathy						
No event (ref)						
≤1 year					−0.0329	0.64
2−5 years					0.0248	
>5 years					−0.0224	
R	0.04	0.17	0.06		0.08	
N	425	87	1757		1757	

*,**,***: significant at 10%, 5%, and 1%, respectively. [a] Additionally adjusted for gender, age at diagnosis, BMI, duration of diabetes, treatment, and history of microvascular complications. [b] Additionally adjusted for gender, age at diagnosis, BMI, duration of diabetes, treatment, and history of macrovascular complications. [c] Additionally adjusted for gender, age at diagnosis, BMI, duration of diabetes, and treatment. [d] We used the *test* command in STATA to check if the estimated coefficients were significantly different.

Table S3. Factors associated with variation in EQ-5D score: results from the Swedish tariff.

**Variable**	**Women**		**Men**		**Total**	
	**Model 1**	**Model 2**	**Model 1**	**Model 2**	**Model 1**	**Model 2**
Constant	0.8692 ***	0.8666 ***	0.9048 ***	0.9050 ***	0.8991 ***	0.8974 ***
Female gender	NA	NA	NA	NA	−0.0266 ***	−0.0260 ***
Age at diagnosis	0.0004	0.0004	0.0008 *	0.0009 *	0.0006 *	0.0006 *
Diabetes duration	−0.0005	−0.0007	−0.0006	−0.0004	−0.0006	−0.0005
BMI	−0.0030 ***	−0.0030 ***	−0.0024 ***	−0.0023 ***	−0.0027 ***	−0.0027 ***
History of macrovascular events	−0.0338 **		−0.0478 ***		−0.0430 ***	
History of microvascular events	−0.0702 ***		−0.0118		−0.0348 **	
AMI history		−0.0003		−0.0182		−0.0119
Stroke history		−0.0551 ***		−0.0635 ***		−0.0590 ***
HF history		−0.0294		−0.0514 **		−0.0422 ***
NAIHD history		−0.0217		−0.0270 **		−0.0249 ***
Kidney disorders history		−0.1036 ***		0.0076		−0.0406*
Retinopathy history		0.0004		−0.0211		−0.0103
Treatment						
Diet (ref)						
OHA	0.0046	0.0068	−0.0080	−0.0084	−0.0009	0.0000
Insulin ± OHA	0.0038	0.0065	−0.0045	−0.0048	0.0009	0.0020
R-squared	0.06	0.07	0.06	0.07	0.06	0.07
N	760	760	997	997	1757	1757

*,**,***: significant at 10%, 5%, and 1%, respectively.

Table S4. Effect of single and multiple events on EQ-5D score: results from the Swedish tariff.

**Variable**	**Model 1**		**Model 2**	
	**Coefficients** [a]	**Equality of coefficients** [b]	**Coefficients** [a]	**Equality of coefficients** [b]
Macrovascular events				
No (ref)				
Single	−0.0314 ***	0.12		
Multiple	−0.0505 ***			
Microvascular events				
No (ref)				
Single	−0.0311	0.84		
Multiple	−0.0364 **			
AMI				
No (ref)				
Single			−0.0113	0.97
Multiple			−0.0121	
Stroke				
No (ref)				
Single			−0.0460 ***	0.05
Multiple			−0.1217 ***	
NAIHD				
No (ref)				
Single			−0.0035	0.05
Multiple			−0.0352 ***	

,*: significant at 5%, and 1%, respectively. [a] Additionally adjusted for gender, age at diagnosis, BMI, duration of diabetes, treatment, history of heart failure, retinopathy, and kidney disorders. [b] We used the test command in STATA to check if the estimated coefficients were significantly different.

Table S5. Effect of diabetes-related complications on EQ-5D scores over time: results from the Swedish tariff.

Variable	Model 1	Model 2	Model 3		Model 4	
	**Coefficients** [a]	**Coefficients** [b]	**Coefficients** [c]	**Equality of coefficients** [d]	**Coefficients** [c]	**Equality of coefficients** [d]
Time since last macrovascular event	0.0032 ***					
Continuous (year)						
No event (ref)						
≤1 year			−0.0479 ***	0.16		
2–5 years			−0.0556 ***			
>5 years			−0.0283 **			
Time since last microvascular event		0.0042				
Continuous (year)						
No event (ref)						
≤1 year			−0.0239	0.76		
2–5 years			−0.0354			
>5 years			−0.0475 **			
Time since last AMI						
No event (ref)						
≤1 year					0.0110	0.42
2–5 years					0.0084	
>5 years					−0.0135	
Time since last stroke						
No event (ref)						
≤1 year					−0.0696 ***	0.44
2–5 years					−0.0848 ***	
>5 years					−0.0429 **	

Table S5. *Cont.*

Variable	Model 1	Model 2	Model 3		Model 4	
	Coefficients [a]	Coefficients [b]	Coefficients [c]	Equality of coefficients [d]	Coefficients [c]	Equality of coefficients [d]
Time since last HF						
No event (ref)						
≤1 year					−0.0114	0.01
2–5 years					−0.1123 ***	
>5 years					−0.0196	
Time since last NAIHD						
No event (ref)						
≤1 year					−0.0290 **	0.88
2–5 years					−0.0318 **	
>5 years					−0.0224 *	
Time since last kidney disorders						
No event (ref)						
≤1 year					−0.0459	0.94
2–5 years					−0.0615 *	
>5 years					−0.0465	
Time since last retinopathy						
No event (ref)						
≤1 year					0.0063	0.52
2–5 years					−0.0046	
>5 years					−0.0358	
R	0.05	0.17	0.07		0.09	
N	425	87	1757		1757	

*,**,***: significant at 10%, 5%, and 1%, respectively. [a] Additionally adjusted for gender, age at diagnosis, BMI, duration of diabetes, treatment, and history of microvascular complications. [b] Additionally adjusted for gender, age at diagnosis, BMI, duration of diabetes, treatment, and history of macrovascular complications. [c] Additionally adjusted for gender, age at diagnosis, BMI, duration of diabetes, and treatment. [d] We used the *test* command in STATA to check if the estimated coefficients were significantly different.

© 2014 by the authors; licensee MDPI, Basel, Switzerland. This article is an open access article distributed under the terms and conditions of the Creative Commons Attribution license (http://creativecommons.org/licenses/by/3.0/).
